# Protecting Nurses from Mistreatment by Patients: A Cross-Sectional Study on the Roles of Emotional Contagion Susceptibility and Emotional Regulation Ability

**DOI:** 10.3390/ijerph18126331

**Published:** 2021-06-11

**Authors:** Bing Liu, Naixin Zhu, Huijuan Wang, Fengyu Li, Chenghao Men

**Affiliations:** School of Management, Shandong University, Jinan 250100, China; liubing@sdu.edu.cn (B.L.); zhunaixin@mail.sdu.edu.cn (N.Z.); huijuan@mail.sdu.edu.cn (H.W.); sdytmch@sdu.edu.cn (C.M.)

**Keywords:** mistreatment by patients, workplace well-being, career commitment, emotional contagion susceptibility, emotional regulation ability

## Abstract

In recent years, patient mistreatment of healthcare workers, especially nurses, has been frequent, endangering the interests of organizations while also threatening nurses’ own development. This study aims to examine from the perspective of nurses’ personal interests whether mistreatment by patients decreases nurses’ workplace well-being and career commitment, and how their susceptibility to emotional contagion and emotional regulation ability might mitigate these negative effects. This study adopted a cross-sectional study design (data were collected through self-reported questionnaires with a two-month time lag between the months of August–October 2017). A total of 289 nurses from three hospitals in Shandong province, China, were recruited to participate in our study. The results reveal that mistreatment by patients is negatively related to nurses’ workplace well-being and career commitment. Emotional contagion susceptibility moderates the relationships between mistreatment by patients and career commitment, while there is no significant buffering effect of mistreatment by patients on workplace well-being. Emotional regulation ability moderates the relationships between mistreatment by patients and both workplace well-being and career commitment. These results suggest that improvements in nurses’ emotional regulation ability and susceptibility to emotional contagion can alleviate the harmful impacts of mistreatment by patients.

## 1. Introduction

“That hurt, you stupid bitch… You sure have a potty mouth… I was prepared to change the dressing and cleanse the wound; what I wasn’t prepared for was the abusive commentary. I managed to get through the tasks, but his hostile comments felt personal, and they devastated me emotionally” [1], this is a statement by a nurse who was verbally abused by a patient. This is not an isolated case; in recent years, verbal violence by patients against nurses in the healthcare industry has been a frequent phenomenon. A recent survey of 4263 nurses in the healthcare industry showed that 54% of respondents had experienced verbal violence by patients [2], including negative emotional behaviors exhibited by patients, such as anger, swearing, insults, yelling, and speaking rudely toward nurses; all of these are part of a phenomenon known as “mistreatment by patients” [3]. These behaviors not only reduce nurses’ work productivity and job performance [4,5] and hinder patient safety [6], but also may affect their own mental health and long-term development. More specifically, mistreatment by patients may directly threaten nurses’ positive attitudes toward their work and careers, which can hinder their career success [7]. It is a big blow to nurses who have spent a lot of time, energy, and money in the early phase and received long-term professional training [8]. Although nurses’ positive affect and attitude are quite valuable for them to keep healthy and achieve good job performance [9,10,11], existing empirical evidence about the impacts of mistreatment by patients on nurses’ positive affective and attitudinal outcomes is still inadequate. Given the prominence of mistreatment by patients in hospitals and the lack of related studies on this topic, and in response to Caldas, Ostermeier, and Cooper’s [12] call for more research into the negative effects of critical incident involvement on healthcare workers, it is worth further investigating the influence of mistreatment by patients on nurses’ personal outcomes. To be specific, the present study will consider two important questions: whether mistreatment by patients affects nurses’ positive affect and attitude toward their work or careers, and how can nurses successfully navigate those stressful mistreatment contexts or experiences?

To answer the first question, our research focused separately on both workplace-related and career-related psychological outcomes of nurses: one is workplace well-being, which includes work-related affect and work satisfaction, representing individuals’ affect and attitudes toward their work [13]; the other is career commitment, which is defined by Blau [14] as “one’s attitude toward their profession or vocation” (p. 280), representing individuals’ attitudes toward their careers. According to affective events theory (AET), affective events experienced in the workplace will affect an individual’s affective reactions and work attitudes [15]. In the healthcare industry, mistreatment by patients is an affective event [16] and is likely to be considered a threat to nursing work [17], thus endangering nurses’ workplace well-being and career commitment. Lower levels of workplace well-being and career commitment prevent employees from reaching their full potential [18] and lead to intentions to change careers [19,20], which may not only hinder their long-term development but also increase nurse turnover and exacerbate nurse shortages. Accordingly, it is theoretically reasonable and practically valuable to pay attention to the influence of patient mistreatment on nurses’ workplace well-being and career commitment.

Furthermore, according to AET, affective reactions mainly include moods and emotions; mistreatment has been demonstrated to be capable of evoking the negative moods and emotions of targets [21]. To prevent mistreatment by patients, the Nursing Regulations (2020) in China notes that those insulting nurses will be economically punished or detained. However, despite these regulations, mistreatment by patients is still frequent [22]. Those mistreatment targets tend to believe that verbal violence by patients is a normal part of the job and therefore choose to tolerate it rather than file accident reports and protect themselves with the regulations [23]. This may lead to a limited preventive effect of the regulations on mistreatment by patients. Therefore, it is important to explore how to reduce the harm caused by mistreatment by patients from the nurses’ perspective. In order to answer this question, we extend our theorizing by identifying the ability to perceive and regulate the negative emotions brought about by mistreatment, both of which may be important for nurses to handle the emotional demands of the nursing work and thus provide good quality care for patients [24,25]. One important individual difference is that, related to the susceptibility to perceive the negative emotion brought about by mistreatment, is emotional contagion susceptibility, which is the tendency to detect and mimic other people’s expressions, movements, postures, and vocalizations, leading to emotional convergence [26]. Another individual ability difference is emotional regulation, which is defined as the ability to regulate one’s own emotions in order to recover rapidly from psychological distress [27]; this is also relevant to consider because individual with high emotional regulation ability can rapidly regulate the negative emotions brought about by mistreatment. Therefore, we explore whether emotional contagion susceptibility and emotional regulation ability can magnify or reduce the effects of mistreatment by patients on workplace well-being and career commitment.

**Figure 1:**
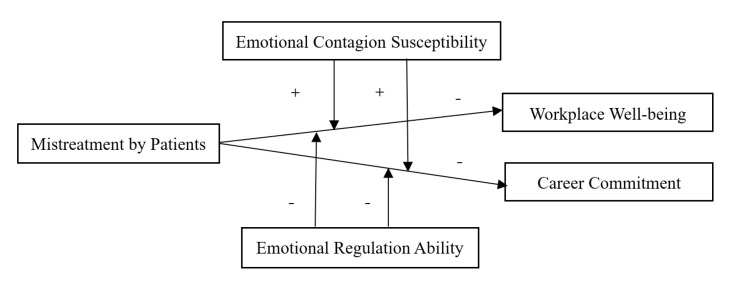
Theoretical Model.

In summary, this study develops and tests a theoretical model regarding the influence of mistreatment by patients on nurses’ workplace well-being and career commitment, and examines the moderating effects of nurses’ emotional contagion susceptibility and emotional regulation ability (shown in Figure 1). Based on this, this study provides three contributions to the literature. First, it contributes to the mistreatment literature by exploring the effects of mistreatment by patients on nurses’ workplace well-being and career commitment in the healthcare industry from the perspective of nurses’ personal interests. Second, by examining whether emotional contagion susceptibility and emotional regulation ability moderate the relationship between mistreatment by patients and nurses’ workplace well-being and career commitment, this study provides an emotion-based perspective to understand when mistreatment by patients is more or less detrimental to nurses. Third, by exploring the buffering effects of nurses’ emotional contagion susceptibility and emotional regulation ability on the negative impacts of mistreatment by patients, this study enriches and improves the boundary conditions of AET, which mainly focused on disposition-related variables and neglected the buffering role played by emotion-related ability.

## 2. Theories and Hypotheses

### 2.1. Mistreatment by Patients, Workplace Well-Being, and Career Commitment

According to AET, events in the workplace significantly affect employees’ affective states and subsequent work attitudes [15]. Nurses’ mistreatment by patients is viewed as a key affective event in the healthcare workplace [16] and research has shown that this kind of incidents can elicit negative moods in employees [28], as well as burnout [29], a decrease in affective commitment [30], and other strong affective reactions [17]. We thus argue that nurses’ workplace well-being and career commitment, which represent the positive attitudes toward their work and careers, respectively [13,14], may also be influenced by being mistreated by patients.

Specifically, mistreatment by a patient can reduce nurses’ workplace well-being in two ways. On the one hand, research has demonstrated that stressful work conditions harm individuals’ positive affect towards work such as joy, happiness, and interest [31]. Considering stress, such as a uncontrollable situation and the possibility of a service goal failure, caused by mistreatment events [16], mistreatment by a patient can lead to the decreased positive affect of nurses, which is an important manifestation of workplace well-being. On the other hand, mistreatment has been viewed as a form of interactional injustice [32], where nurses who experience interactional injustice from patients are less likely to continue to feel satisfied with their work [33]. Patients who mistreat nurses while they are working make nurses feel that their work is full of unfriendliness and that they are tired of the working environment of nursing. As such, it is difficult for them to obtain a sense of pleasure and achievement in their work. This was also supported by Hershcovis and Barling’s [34] finding that aggression in the workplace has an adverse effect on employees’ satisfaction with their job. Therefore, when nurses suffer mistreatment by patients, they experience lower levels of workplace well-being. Considering these points, we propose the following.

**Hypothesis** **1.** *Mistreatment by patients is negatively related to workplace well-being*.

Nurses have to spend a great deal of time, energy, and money preparing for their chosen career [8]. Once in the nursing career, they are often faced with a heavy workload, low nurse–patient ratios, long working hours, night shifts, and underpayment [35]. When nurses devote themselves wholeheartedly to the care of their patients only to be the objects of those patients’ mistreatment [17], it may make them feel that their efforts are not equal to the rewards of their profession. Thus, their willingness to offer the best nursing care possible and their perception of their career value may be reduced, in turn lowering their commitment to nursing work. We therefore propose the following.

**Hypothesis** **2.** *Mistreatment by patients is negatively related to career commitment*.

### 2.2. The Moderating Role of Emotional Contagion Susceptibility

Emotional contagion relates to the transfer of emotional states between individuals, leading to the observer’s emotional state corresponding to that of the sender [26]. Previous research has suggested that there are individual differences when people are infected by others’ emotions [36]. Individuals who are more susceptible to emotional contagion will suffer stronger effects of emotional contagion [37]. Patients tend to engage in mistreatment behavior toward nurses in order to vent their negative emotions, which may be aroused by long waiting times, a patient’s situation, or other problems [38]. When patients express negative emotions to nurses (e.g., anger, anxiety, etc.), it may cause nurses to experience a congruent negative emotional state. However, nurses with different emotional contagion susceptibilities may react differently to these affect stimuli. In this study, we propose that the susceptibility of nurses to emotional contagion may strengthen the negative effects of mistreatment by patients on nurses’ workplace well-being and career commitment for two reasons.

First, individuals who are susceptible to emotional contagion are more likely to catch a sender’s negative emotions [39]. According to AET, mistreatment by patients, as an affective event, may affect employees’ attitudes and emotions [15]. Nurses who are susceptible to the negative emotional contagion process involved in the mistreatment by a patient will more readily experience negative emotional states that are consistent with those of the patients [36], and have more intense negative emotions after being mistreated because of the patients’ emotions. In this case, their workplace well-being (e.g., job satisfaction and positive affect) and career commitment tend to be damaged. Second, nurses who are susceptible to emotional contagion are more likely to imitate the negative facial expressions and abusive tone adopted by patients who are mistreating them [40]. Yet, the display rules for patient care obviously do not allow nurses to express and vent these grievances and emotions [3]. In other words, nurses need to suppress the negative emotional expressions and behaviors caused by automatic imitation, which makes it difficult for nurses to maintain a positive attitude toward their work and careers. Therefore, we hypothesize the following.

**Hypothesis** **3.** *Emotional contagion susceptibility moderates the negative effect of mistreatment by patients on workplace well-being, such that the negative relationship is stronger when individuals’ emotional contagion is high rather than low*.

**Hypothesis** **4.** *Emotional contagion susceptibility moderates the negative effect of mistreatment by patients on career commitment, such that the negative relationship is stronger when individuals’ emotional contagion is high rather than low*.

### 2.3. The Moderating Role of Emotional Regulation Ability

Previous research has shown that the ability to regulate emotions can help people to adjust their emotional responses to negative events [41]. Logically, therefore, nurses’ ability to regulate their emotions can help them to more successfully address the issue of mistreatment by patients. Put differently, nurses’ emotional regulation abilitiy may attenuate the negative effects of mistreatment by patients on their workplace well-being and career commitment, and the reasons are twofold.

First, unlike nurses who have low levels of emotional regulation ability, those with a higher level of emotional regulation ability are likely to reappraise negative mistreatment events; that is, they are able to turn the negative implications of mistreatment into positives [42,43]. For example, nurses with high levels of emotional regulation ability would treat being mistreated by patients as emotional venting [44], rather than as an act specifically targeting them, which will not affect nurses’ recognition of the meaning of their work. In this case, they will be less likely to make a negative evaluation of their work after suffering mistreatment by a patient. Second, nurses with strong emotional regulation can more easily solve those mistreatment problems with patience and rapidly engage in nursing work [45,46]. Owing to the capability to quickly adjust their emotions and express them in a suitable way, nurses with high levels of emotional regulation are less likely to treat mistreatment by patients as a threat [47], and are able to meet the display rules of their organization [48]. In this case, the possibility of their decreased workplace well-being and career commitment resulting from mistreatment by patients will be reduced. We thus propose the following.

**Hypothesis** **5.** *Emotional regulation ability moderates the negative effects of mistreatment by patients on workplace well-being, such that the negative relationship is weaker when individuals’ emotional regulation ability is high rather than low*.

**Hypothesis** **6.** *Emotional regulation ability moderates the negative effects of mistreatment by patients on career commitment, such that the negative relationship is weaker when individuals’ emotional regulation ability is high rather than low*.

## 3. Materials and Methods

### 3.1. Participants and Procedure

We collected data from nurses working in frontline departments at three different hospitals located in Shandong province, China. The data collection was conducted from August to October 2017. The inclusion criteria for hospitals were large public hospitals with more than 200 beds, which receive quite a lot of patients every day, and mistreatment by patients will happen more often in these hospitals. We explained to the human resource managers of the final three selected hospitals the purpose and procedures of the study to obtain permission and seek their support. With the help of human resource managers at the three hospitals, we coded the questionnaires confidentially and distributed them to the nurses after they were off duty. A cross-sectional study design was used in our study. To minimize common method variance, we followed Podsakoff, Mackenzie, Lee, and Podsakoff’s [49] recommendations and collected two waves of data with a two-month time lag. Participants provided demographic information (e.g., age, tenure, education level) and completed measures of mistreatment by patients and emotional contagion susceptibility at time 1. Participants completed measures of emotional regulation ability, workplace well-being, and career commitment at time 2. We matched the collected data via an anonymous code. In order to maximize the response rate, each participant was asked to participate voluntarily and was compensated with a small gift after participation; all were assured that their personal information would be kept confidential.

The total number of nurses in the three hospitals was 1458. However, due to the voluntary nature of the study, at time 1, we distributed questionnaires to 499 nurses and received 491 responses (98.40% response rate). As participants left the hospitals or responses were excluded due to missing data or invalid information, 451 participants were left for the second stage. At time 2, 356 nurses responded (78.9% response rate), with 67 excluded due to missing data or invalid information and 289 being retained in our analyses. Among the 289 matched responses, the mean age of the participants was 31.32 years, the mean job tenure was 9.18 years, 98% of the participants were female, and 71.28% of the participants held a bachelor’s degree.

### 3.2. Measures

The measures used were originally published in English, so we used the translation-back-translation procedure proposed by Brislin [50] to ensure the equivalence of the Chinese version of the questionnaire, and used a five-point Likert scale to assess each item.

#### 3.2.1. Mistreatment by Patients

Mistreatment by patients was measured using an eight-item scale adapted from Skarlicki et al. [51]. A sample item is: “Patients spoke aggressively to me” (1 = never, 5 = frequently; α = 0.878).

#### 3.2.2. Workplace Well-Being 

Workplace well-being was measured using six items from the subscale of Zheng et al.’s [13] employee well-being scale. A sample item is: “I find real enjoyment in my work” (1 = strongly disagree, 5 = strongly agree; α = 0.902). 

#### 3.2.3. Career Commitment 

Career commitment was measured using six items adapted from Blau [48] and Kim, Um, Kim, and Kim [49]. A sample item includes: “This is the ideal profession for a life work” (1 = strongly disagree, 5 = strongly agree; α = 0.898).

#### 3.2.4. Emotional Contagion Susceptibility 

Emotional contagion susceptibility was assessed using the three-item scale of McBane [52]. A sample item is: “I become nervous if others around me are nervous” (1 = strongly disagree, 5 = strongly agree; α = 0.659). 

#### 3.2.5. Emotional Regulation Ability 

Emotional regulation ability was assessed using the four-item emotional regulation subscale of the emotional intelligence scale [27]. A sample item is: “I am able to control my temper so that I can handle difficulties rationally” (1 = strongly disagree, 5 = strongly agree; α = 0.879).

There is evidence suggesting that demographic information is relevant to affective and attitudinal variables [53,54]; we thus controlled for the effects of nurses’ gender, age, education level, and job tenure. Gender was coded as 0 (male) or 1 (female). Age and job tenure were self-reported in years. Education level was coded into four categories (1 = employees had completed a junior college degree or below; 2 = employees held a college degree; 3 = employees held a bachelor’s degree; 4 = employees held a postgraduate degree or above).

### 3.3. Data Analysis

Data analysis was performed using SPSS for Windows Version 20.0, and Mplus 8.3 was used for this study. Reliability, factor loading, and descriptive statistics were conducted using SPSS. Harman’s one-factor test was implemented to evaluate the common method variance using SPSS, and confirmatory factor analysis were conducted to test the suitability of the measurement model using Mplus. We used Mplus to test our hypotheses. We implemented a multiple regression analysis to explore the relationships between the research variables, and simple slope analysis was performed to further investigate the interaction effects. A two-tailed *p* < 0.05 was considered statistically significant.

### 3.4. Ethics Considerations

The study was approved by the Institutional Review Board (protocol code: 2017-0601-05). All participants were given the informed consent statement before participating in the study, and were aware that they could withdraw participation any time during the study. To protect the privacy of the participants, all questionnaire responses were handled uniformly under a unique personal code.

## 4. Results

### 4.1. Descriptive Statistics

Table 1 presents the descriptive statistics and correlations of the variables for this study. Mistreatment by patients was negatively correlated with workplace well-being (*r* = −0.210, *p* < 0.001) and career commitment (*r* = −0.161, *p* < 0.01).

### 4.2. Harman’s One-Factor Test

Since the data on mistreatment by patients, workplace well-being, career commitment, emotional contagion susceptibility, and emotional regulation ability were collected from the same source, we conducted Harman’s one-factor test to evaluate common method variance (CMV) through the unrotated factor analysis. According to the results, the first factor explained only 23.45% of the variance, below the critical value of 40%. These findings suggested that common method variance was not a significant problem in this research [49].

### 4.3. Confirmatory Factor Analysis

Before testing the hypothesized relationships, we conducted a confirmatory factor analysis (CFA) with a maximum likelihood estimation to ensure the validity of the outcome variable and the distinct factor structure of the key variables. The results indicated that our hypothesized five-factor model separating mistreatment by patients, emotional contagion susceptibility, emotional regulation ability, workplace well-being, and career commitment factors fit the data best (χ^2^ = 593.080, df = 314, *p* < 0.01, comparative fit index (CFI) = 0.930, Tucker–Lewis index (TLI) = 0.922, root mean square error of approximation (RMSEA) = 0.055, standardized root mean square residual (SRMR) = 0.048) and fit better than alternative models (e.g., a four-factor model that combined workplace well-being and career commitment, χ^2^ = 976.007, df = 318, *p* < 0.01, CFI = 0.835, TLI = 0.818, RMSEA = 0.085, SRMR = 0.064; a three-factor model that combined mistreatment by patients, emotional contagion susceptibility, emotional regulation ability, χ^2^ = 1350.294, df = 321, *p* < 0.01, CFI = 0.743, TLI = 0.718, RMSEA = 0.105, SRMR = 0.124; a two-factor model that combined emotional contagion susceptibility, emotional regulation ability, workplace well-being, and career commitment, χ^2^ = 1597.427, df = 323, *p* < 0.01, CFI = 0.681, TLI = 0.654, RMSEA = 0.117, SRMR = 0.097; a one-factor model that combined mistreatment by patients, emotional contagion susceptibility, emotional regulation ability, workplace well-being, and career commitment, χ^2^ = 2449.089, df = 324, *p* < 0.01, CFI = 0.468, TLI = 0.424, RMSEA = 0.151, SRMR = 0.151). Therefore, the empirical distinctiveness of the variables used in our research was supported.

### 4.4. Hypothesis Testing

We used Mplus 8.3 to test the main effects and moderation effects. We tested Hypothesis 1 by regressing workplace well-being on mistreatment by patients, emotional contagion susceptibility, emotional regulation ability, and control variables (i.e., gender, age, education, tenure) (Model 1). The results, as shown in Model 1 in Table 2, indicated that mistreatment by patients had a significantly negative effect on workplace well-being (β = −0.189, *p* < 0.05). We tested Hypothesis 2 by regressing career commitment on mistreatment by patients, emotional contagion susceptibility, emotional regulation ability, and control variables (i.e., gender, age, education, tenure) (Model 4). The results indicated that mistreatment by patients had a significant negative effect on career commitment (β = −0.158, *p* < 0.05). Therefore, Hypotheses 1 and 2 were supported.

In our analyses of moderation effects, we centered the predictors (i.e., mistreatment by patients, emotional contagion susceptibility, and emotional regulation ability) by their grand means and created the interaction terms using the mean-centered scores. We tested Hypothesis 3 by adding the interaction effects between mistreatment by patients and emotional contagion susceptibility on workplace well-being (Model 2). However, as shown in Model 2 in Table 2, the interaction of mistreatment by patients with emotional contagion susceptibility was not significant in predicting workplace well-being (β = 0.003, n.s.). Thus, Hypothesis 3 was not supported.

**Figure 2:**
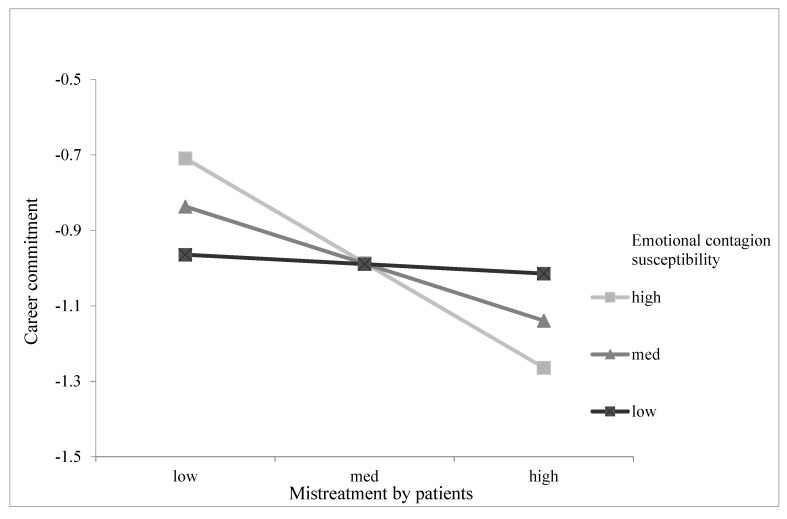
Interaction between mistreatment by patients and emotional contagion susceptibility on career commitment.

We tested Hypothesis 4 by adding the interaction effects between mistreatment by patients and emotional contagion susceptibility on career commitment (Model 5). Results (see Model 5 in Table 2) showed that the interaction of mistreatment by patients with emotional contagion susceptibility was significant in predicting career commitment (β = −0.293, *p* < 0.05). Based on Aiken and West’s [55] study, we used the simple slope analyses at high and low levels of emotional contagion susceptibility (1 SD above and below the mean) to provide additional evidence. Figure 2 shows the interaction plots, indicating that the negative relationship between mistreatment by patients and career commitment was stronger when the level of emotional contagion susceptibility was high. Simple slope tests (see Table 3) showed that the relationship between mistreatment by patients and career commitment was significantly negative for individuals with a high level of emotional contagion susceptibility (β = −0.426, *p* < 0.01) but not significant for individuals with a low level of emotional contagion susceptibility (β = 0.010, n.s.); the difference of the indirect effects between the two conditions was significant (β = −0.437, *p* < 0.05). Therefore, Hypothesis 4 was supported.

**Figure 3:**
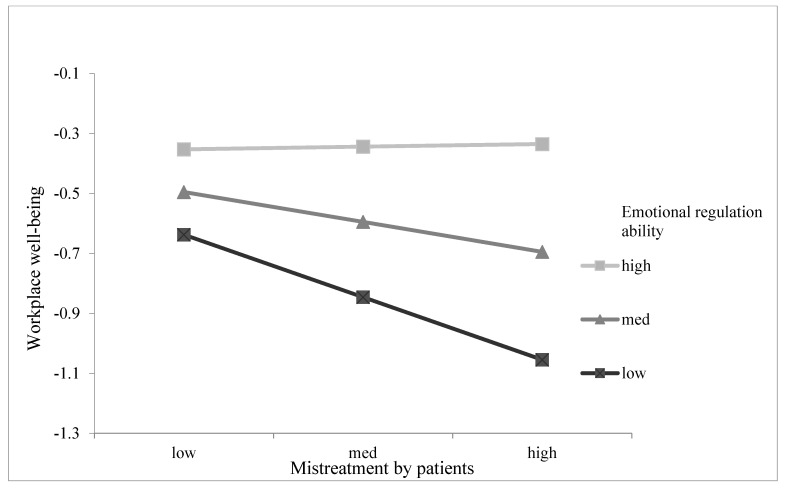
Interaction between mistreatment by patients and emotional regulation ability on workplace well-being.

We tested Hypothesis 5 by adding the interaction effects between mistreatment by patients and emotional regulation ability on workplace well-being (Model 3). As illustrated in Model 3 in Table 2, the interaction of mistreatment by patients with emotional regulation ability was significant in predicting workplace well-being (β = 0.239, *p* < 0.05). As shown in Figure 3, the negative relationship between mistreatment by patients and workplace well-being was weaker at high levels of emotional regulation ability. As shown in Table 3, the relationship between mistreatment by patients and workplace well-being was significantly negative for individuals with a low level of emotional regulation ability (β = −0.359, *p* < 0.001) but not significant for individuals with a high level of emotional regulation ability (β = 0.012, n.s.); the difference of the indirect effects between the two conditions was significant (β = 0.371, *p* < 0.05). These results provided support for Hypothesis 5.

**Figure 4:**
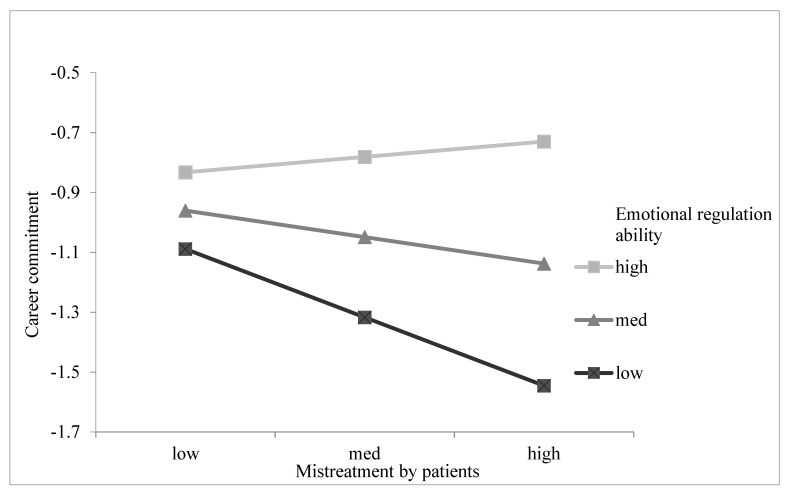
Interaction between mistreatment by patients and emotional regulation ability on career commitment.

We tested Hypothesis 6 by adding the interaction effects between mistreatment by patients and emotional regulation ability on career commitment (Model 6). Results (see Model 6 in Table 2) showed that the interaction of mistreatment by patients with emotional regulation ability was significant in predicting career commitment (β = 0.294, *p* < 0.05). Figure 4 shows the interaction plots, which are consistent with our expectation: when the level of emotional regulation ability was high, the negative relationship between mistreatment by patients and career commitment was weaker. As shown in Table 3, the relationship between mistreatment by patients and career commitment was significantly negative for individuals with a low level of emotional regulation ability (β = −0.367, *p* < 0.001), but not significant for individuals with a high level of emotional regulation ability (β = 0.090, n.s.); the difference of the indirect effects between the two conditions was significant (β = 0.458, *p* < 0.05). Therefore, Hypothesis 6 was supported.

## 5. Discussion

This study aimed to investigate the impacts of mistreatment by patients on nurses themselves and how nurses can mitigate these negative impacts by improving their own abilities. Our results show that mistreatment by patients endangers nurses’ workplace well-being and career commitment. The negative effects of mistreatment by patients on nurses’ career commitment are weaker when nurses have low (vs. high) susceptibility to emotional contagion. Unexpectedly, the moderation effect of emotional contagion susceptibility on the relationship between mistreatment by patients and workplace well-being is not supported in our study. One possible explanation is that nurses who are susceptible to emotional contagion are also better able to empathize with others; that is, they may perceive not only the patients’ anger but also their worry and fears [36], thus forgiving them for their mistreatment and alleviating the negative feelings toward the current job brought about by their being mistreated. Therefore, nurses’ perceived workplace well-being may not decrease after suffering mistreatment, at least in the case of nurses with high susceptibility to emotional contagion. Moreover, our results suggest that the negative effects of mistreatment by patients on nurses’ workplace well-being and career commitment are both weaker when nurses have high (vs. low) emotional regulation abilities.

### 5.1. Theoretical Implications

Our study makes several key theoretical implications to existing literature. First, by demonstrating the negative influence of mistreatment by patients on nurses’ workplace well-being and career commitment, our study broadens researchers’ understanding of the consequences of mistreatment by patients. Consistent with previous research demonstrating that workplace violence negatively affects nurses [56], our findings suggest that mistreatment by patients harms nurses’ workplace well-being and career commitment. According to Cheung, Lee, and Yip’s study [57], verbal violence in the workplace is more prevalent than physical assault in healthcare settings, and should not be considered less serious than physical assault, and therefore worth more attention. Although numerous extant studies have examined the impacts of mistreatment by patients on nurses’ behavioral outcomes, such as work productivity and job performance [4,5], and negative affective outcomes, such as burnout and depression [29,58], little attention has been paid to their positive affective and attitudinal outcomes. However, we think exploring the impact of mistreatment by patients on nurses’ positive psychology-related outcomes (e.g., workplace well-being and career commitment) is particularly important because the traumatic work environment requires nurses to have a positive attitude towards their work and career to maintain their mental health and achieve development. Especially, we paid attention to the outcomes related to individuals’ long-term development, namely career commitment, thus expanding the existing research on the outcome of mistreatment event that mainly focused on the targets’ attitudes and behaviors within the workplace [34]. All in all, by focusing on mistreatment behaviors and providing rigorous empirical evidences on the detrimental impacts of mistreatment by patients on nurses’ workplace-related and career-related outcomes (i.e., workplace well-being and career commitment), this study provides an integrated perspective to understand the consequences of mistreatment by patients on nurses and responds to previous researchers’ calls for more studies on the negative influence of health care workers’ involvement in critical incidents [12].

Second, our study provides new insights into the boundary conditions of the influence of mistreatment by patients on nurses’ workplace well-being and career commitment by revealing when mistreatment by patients is more or less harmful to nurses from an emotion-based perspective (in terms of emotional contagion susceptibility and emotional regulation ability). Although several existing studies have explored the buffering effects of some emotion-based variables on the negative impacts of mistreatment behavior [3,59], most of them tend to focus on the buffering role played by individuals’ emotion-related traits or strategies, ignoring their personal ability for emotional perception and regulation, which is easier to improve through training. As the negative emotions of nurses can affect nursing care and thus jeopardize patient safety [60], we believe exploring individual differences in nurses’ perceptions of others’ emotions and control of their own emotions, which are reflected as susceptibility to emotional contagion and emotional regulation, is necessary for mitigating the negative influence of mistreatment by patients. Consistent with previous research, our study shows that individuals more susceptible to emotional contagion will suffer stronger consequences of emotional contagion [61]. That is, nurses with high susceptibility to emotional contagion will doubt the meaningfulness of the nursing profession after experiencing mistreatment by patients and therefore decrease their career commitment. In addition, our results suggest that the extent to which nurses are negatively affected by mistreatment can be influenced by their abilities to regulate their emotions, confirming previous researchers’ findings that emotional regulation can buffer the negative effects of negative events [62]. By amplifying and attenuating the effects of nurses’ emotional contagion susceptibility and emotional regulation ability in the relationship between mistreatment by patients and positive psychological outcomes (i.e., workplace well-being and career commitment in the present study), we deepen the understanding of the boundary conditions of mistreatment by patients on nurses’ affect and attitude from emotion-based perspectives. 

Third, our study expands the scope of AET by exploring the moderating roles of emotional contagion susceptibility and emotional regulation ability in influencing the detrimental effects of mistreatment by patients, which is considered as an important affective event. Weiss and Cropanzano [15] illustrate that an individual’s dispositions can affect the influences of affective events on their affective reactions and work attitudes. Existing studies drawing on AET have mainly examined the moderating effects of disposition-related variables, such as negative affectivity and hostility [63,64]; some also explored the boundary conditions of leadership styles, emotional regulation styles, etc. [65,66], but they ignored the possible moderating effect of emotional-related ability. Our study focuses on the central role of emotions in AET, suggesting that the extent to which one’s ability to control emotions without being influenced by others can modify the intensity of emotions brought about by an affective event, thus enriching and improving AET.

### 5.2. Practical Implications

Our study has important implications for nurses themselves and the nursing practice. First, mistreatment was found to have more harmful effects on those who are susceptible to emotional contagion or those who have low levels of emotional regulation ability. In contrast, individuals who are less likely to be affected by others’ emotions and who are able to regulate their negative emotions may mitigate the consequences of mistreatment. Therefore, to prevent nurses from being affected by mistreatment by patients, it is imperative to improve individuals’ ability to manage their own emotions by providing training programs. In some Chinese hospitals, training programs on mistreatment by patients are not well developed, and mostly focus on nurse–patient communication. While these courses can somewhat reduce the probability of mistreatment by patients [67], they do not address how nurses can recover from mistreatment by patients. Supervisors should provide training programs that can impart knowledge of emotional management and emotional blueprint training [56] to improve nurses’ emotional regulation abilities in order to facilitate nurses’ recovery from mistreatment by patients. Nurses should actively participate in these training programs and apply relevant skills or proactively seek the help of colleagues or supervisors when a mistreatment event occurs. In addition, by focusing on emotions, our study indicates that it is necessary for supervisors to pay attention to nurses’ emotional states, especially those who are susceptible to the emotions of others. Supervisors should provide nurses with support when they are in a poor emotional state, especially following mistreatment by patients. They can do this by, for example, communicating with nurses and providing psychological counseling. 

Second, due to the serious negative effects of mistreatment by patients on nurses’ attitudes toward their work and careers, measures should be adopted to prevent patients from engaging in mistreatment behavior. Previous research suggests that patients engage in mistreatment for a variety of reasons: as a result of misunderstandings [68], anger caused by their situation, long waiting times, and the enforcement of hospital policies [38]. To control mistreatment events, it is necessary for supervisors to adopt a series of preventive measures. They should develop a climate of mutual respect [69] and encourage nurses to actively communicate with patients in order to avoid mistreatment caused by misunderstandings and concerns about a patient’s condition. To reduce mistreatment events caused by dissatisfaction with a hospital’s policies or practices, supervisors could also put up clear notices around their hospital to make sure patients know exactly how to assert their rights. Meanwhile, the probability of medical errors may increase with lower workplace well-being and career commitment of nurses [70], which makes it important for hospital managers to be more concerned about the workplace well-being and career commitment of the nursing population, especially those who have suffered from mistreatment. Specifically, supervisors should pay attention to praising nurses for their performance and achievements, and provide them with regular career counseling services to maintain and enhance their workplace well-being and career commitment [71].

### 5.3. Limitations and Future Research Directions

Although the current study makes several significant theoretical contributions, some limitations that point to promising future research directions are worth noting. First, our data rely on self-reporting. Although the Harman’s one-factor test and CFA conducted in our study showed that common method variance was not a serious problem, future research may benefit from collecting data from multiple sources to minimize the bias of the self-report method.

Second, our study was conducted with a sample of nurses in Shandong province of China, which may limit the generalizability of our findings. Since individuals in different cultures may differ in their responses to mistreatment behaviors [72], future testing of our findings in the context of other countries and regions may be a fruitful direction. Moreover, our data were collected before the COVID-19 pandemic. This pandemic may lead to a dramatic increase in the number of patients, a surge in hospital stress, and more mistreatment behaviors by patients. Future research could examine whether there are more mistreatment behaviors imposed by patients in the context of the COVID-19 pandemic and whether there are more serious consequences of mistreatment by patients in a pandemic context.

Third, our study only focuses on the relationship between mistreatment by patients and nurses’ workplace well-being and career commitment. However, our study did not discuss the subsequent impact of mistreatment by patients on the long-term development of nurses’ careers, which is significant for nurses who spend a lot of time, energy, and money preparing for a nursing career [8]. It may be meaningful for future longitudinal studies to explore the subsequent impacts of mistreatment by patients on nurses’ long-term development, such as career success, through workplace well-being and career commitment.

Fourth, our study mainly focuses on the moderating effects of emotional contagion susceptibility and emotional regulation ability to capture the influences of individuals’ emotion-based abilities on the relationships between mistreatment by patients and its consequences. This is by no means comprehensive. Given that conversations with colleagues may alleviate the negative emotions caused by mistreatment by patients, future research may also benefit from considering the buffer effects of colleague-related factors, such as communication with and support from colleagues.

Fifth, hospital pressure rates may also be a critical antecedent worth considering. In hospitals with high pressure, nurses are required to handle more patients and work, and therefore may be more responsive to mistreatment by patients [5]. Therefore, we suggest future research to further explore the important effect of hospital pressure rates on nurses’ reactions toward mistreatment by patients.

## 6. Conclusions

Mistreatment by patients is a common phenomenon that occurs between patients and nurses. This study confirms that nurses’ workplace well-being and career commitment will be significantly reduced after suffering mistreatment by patients. Nurses’ susceptibility to emotional contagion can aggravate the negative impact of mistreatment by patients on career commitment. Nurses’ emotional regulation ability can alleviate the negative impacts of mistreatment by patients on workplace well-being and career commitment. Although the moderating effect of nurses’ susceptibility to emotional contagion on the relationship between mistreatment by patients and workplace well-being has not been demonstrated, considering that emotional contagion susceptibility exacerbates the effect of mistreatment by patients on career commitment, we should still pay attention to nurses’ susceptibility to emotional contagion. Therefore, according to our findings, we recommend that nursing administrators intervene to improve nurses’ emotional regulation ability and reduce their susceptibility to emotional contagion in order to avoid being affected by mistreatment.

## Figures and Tables

**Table 1:** Means, standard deviation, and correlations between study variables.

	1	2	3	4	5	6	7	8	9
1. Gender									
2. Age	0.112								
3. Education	0.053	0.115							
4. Tenure	0.143 *	0.919 ***	0.076						
5. Mistreatment by patients	−0.037	−0.086	−0.207 ***	−0.085	(0.878)				
6. Workplace well-being	−0.072	0.119 *	0.143 *	0.082	−0.210 ***	(0.902)			
7. Career commitment	−0.058	0.039	0.127 *	−0.017	−0.161 **	0.598 ***	(0.898)		
8. Emotional contagion susceptibility	0.063	0.023	0.115 *	0.020	−0.103	−0.082	0.045	(0.659)	
9. Emotional regulation ability	−0.027	−0.043	0.013	−0.064	−0.083	0.355 ***	0.314 ***	−0.158 **	(0.879)
Mean	0.98	31.310	2.700	9.180	1.620	3.179	2.884	3.269	3.108
S. D.	0.143	5.826	0.537	6.319	0.598	0.703	0.858	0.746	0.777

Notes: N = 289. Reliabilities are in parentheses. * *p* < 0.05, ** *p* < 0.01, *** *p* < 0.001.

**Table 2:** Results of hypotheses testing.

	Workplace Well-Being			Career Commitment		
Predictor	Model 1	Model 2	Model 3	Model 4	Model 5	Model 6
Intercept	2.080 ** (0.679)	−1.575 * (0.625)	−0.446 (0.579)	0.791 (0.790)	−1.975 ** (0.726)	−1.257 (0.671)
Gender	−0.399 (0.245)	−0.398 (0.245)	−0.381 (0.243)	−0.337 (0.362)	−0.350 (0.361)	−0.316 (0.359)
Age	0.025 (0.022)	0.025 (0.022)	0.025 (0.020)	0.042 (0.023)	0.038 (0.023)	0.042 * (0.020)
Education	0.131 (0.072)	0.131 (0.072)	0.129 (0.071)	0.132 (0.084)	0.136 (0.085)	0.130 (0.083)
Tenure	−0.010 (0.020)	−0.010 (0.020)	−0.011 (0.018)	−0.036 (0.021)	−0.036 (0.021)	−0.037 * (0.019)
Mistreatment by patients (MP)	−0.189 * (0.074)	−0.188 * (0.076)	−0.173 * (0.070)	−0.158 * (0.076)	−0.208 * (0.084)	−0.138 (0.076)
Emotional contagion susceptibility (ECS)	−0.052 (0.056)	−0.051 (0.056)	−0.057 (0.056)	0.087 (0.077)	0.062 (0.073)	0.080 (0.075)
MP×ECS		0.003 (0.100)			−0.293 * (0.134)	
Emotional regulation ability (ERA)	0.301 *** (0.055)	0.301 *** (0.055)	0.314 *** (0.057)	0.341 *** (0.066)	0.344 *** (0.066)	0.357 *** (0.067)
MP×ERA			0.239 * (0.118)			0.294 * (0.125)
R^2^	0.193 ***	0.193 ***	0.211 ***	0.148 **	0.168 ***	0.166 ***

Notes: N = 289. Unstandardized coefficients are presented. Standard errors are reported in parentheses. * *p* < 0.05, ** *p* < 0.01, *** *p* < 0.001.

**Table 3:** Results of simple slope analyses.

Emotional Contagion Susceptibility					Emotional Regulation Ability			
Moderation effect		Estimate	SE	95% CI	Moderation effect	Estimate	SE	95% CI
Workplace well-being	High emotional contagion susceptibility (mean +1 SD)	−0.186	0.114	[−0.410, 0.040]	High emotional regulation ability (mean +1 SD)	0.012	0.128	[−0.234, 0.266]
	Low emotional contagion susceptibility (mean −1 SD)	−0.191	0.098	[−0.370, 0.014]	Low emotional regulation ability (mean −1 SD)	−0.359 ***	0.101	[−0.546, −0.153]
	The difference	0.005	0.149	[−0.293, 0.294]	The difference	0.371 *	0.183	[0.027, 0.754]
Career commitment	High emotional contagion susceptibility (mean +1 SD)	−0.426 **	0.129	[−0.679, −0.167]	High emotional regulation ability (mean +1 SD)	0.090	0.143	[−0.163, 0.393]
	Low emotional contagion susceptibility (mean −1 SD)	0.010	0.132	[−0.204, 0.308]	Low emotional regulation ability (mean −1 SD)	−0.367 ***	0.100	[−0.552, −0.156]
	The difference	−0.437 *	0.200	[−0.848, −0.076]	The difference	0.458 *	0.194	[0.108, 0.878]

Notes: N = 289. * *p* < 0.05, ** *p* < 0.01, *** *p* < 0.001.

## Data Availability

Data are available on request due to ethical restrictions. Due to the confidentiality of the respondents, the data are not publicly available.

## References

[1] Stopping Verbally Abusive Patients in Their Tracks. https://canadian-nurse.com/en/articles/issues/2012/may-2012/stopping-verbally-abusive-patients-in-their-tracks.

[2] Nursing Trends and Salary Survey Results 2020. https://www.myamericannurse.com/2020-nursing-trends-and-salary-survey-results/.

[3] Goussinsky R., Livne Y. (2016). Coping with Interpersonal Mistreatment: The Role of Emotion Regulation Strategies and Supervisor Support. J. Nurs. Manag..

[4] Liu J., Zheng J., Liu K., Liu X., Wu Y., Wang J., You L. (2019). Workplace Violence against Nurses, Job Satisfaction, Burnout and Patient Safety in Chinese Hospitals. Nurs. Outlook.

[5] Lin W.-Q., Wu J., Yuan L.-X., Zhang S.-C., Jing M.-J., Zhang H.-S., Luo J.-L., Lei Y.-X., Wang P.-X. (2015). Workplace Violence and Job Performance among Community Healthcare Workers in China: The Mediator Role of Quality of Life. Int. J. Environ. Res. Public Health.

[6] Kim K. (2020). Exploring the Influence of Workplace Violence and Bystander Behavior on Patient Safety in Korea: A Pilot Study. J. Nurs. Manag..

[7] Gunawan J., Aungsuroch Y., Fisher M., Marzilli C., Liu Y. (2020). Factors Related to the Clinical Competence of Registered Nurses: Systematic Review and Meta-Analysis. J. Nurs. Scholarsh..

[8] Alameddine M., Baroud M., Kharroubi S., Hamadeh R., Ammar W., Shoaib H., Khodr H. (2017). Investigating the Job Satisfaction of Healthcare Providers at Primary Healthcare Centres in Lebanon: A National Cross-Sectional Study. Health Soc. Care Community.

[9] González-Gancedo J., Fernández-Martínez E., Rodríguez-Borrego M.A. (2019). Relationships among General Health, Job Satisfaction, Work Engagement and Job Features in Nurses Working in a Public Hospital: A Cross-Sectional Study. J. Clin. Nurs..

[10] Motlagh F., Nobahar M., Raiesdana N. (2020). The Relationship of Moral Intelligence and Social Capital with Job Satisfaction among Nurses Working in the Emergency Department. Int. Emerg. Nurs..

[11] Lotfi Z., Atashzadeh-Shoorideh F., Mohtashami J., Nasiri M. (2018). Relationship between Ethical Leadership and Organisational Commitment of Nurses with Perception of Patient Safety Culture. J. Nurs. Manag..

[12] Caldas M., Ostermeier K., Cooper D. (2020). When Helping Hurts: COVID-19 Critical Incident Involvement and Resource Depletion in Health Care Workers. J. Appl. Psychol..

[13] Zheng X., Zhu W., Zhao H., Zhang C. (2015). Employee Well-Being in Organizations: Theoretical Model, Scale Development, and Cross-Cultural Validation. J. Organ. Behav..

[14] Blau G. (1985). The Measurement and Prediction of Career Commitment. J. Occup. Psychol..

[15] Weiss H., Cropanzano R. (1996). Affective Events Theory: A Theoretical Discussion of The Structure, Cause and Consequences of Affective Experiences at Work. Res. Organ. Behav..

[16] Baranik L.E., Wang M., Gong Y., Shi J. (2017). Customer Mistreatment, Employee Health, and Job Performance: Cognitive Rumination and Social Sharing as Mediating Mechanisms. J. Manag..

[17] Karaeminogullari A., Erdogan B., Bauer T. (2018). Biting the Hand That Heals: Mistreatment by Patients and the Well-Being of Healthcare Workers. Pers. Rev..

[18] Singhal H., Rastogi R. (2018). Psychological Capital and Career Commitment: The Mediating Effect of Subjective Well-Being. Manag. Decis..

[19] Chang H.-Y., Chu T.-L., Liao Y.-N., Chang Y.-T., Teng C.-I. (2019). How Do Career Barriers and Supports Impact Nurse Professional Commitment and Professional Turnover Intention?. J. Nurs. Manag..

[20] Wilson V., Donsante J., Pai P., Franklin A., Bowden A., Almeida S. (2021). Building Workforce Wellbeing Capability the Findings of a Wellness Self-care Program. J. Nurs. Manag..

[21] Koopmann J., Wang M., Liu Y., Song Y. (2015). Customer Mistreatment: A Review of Conceptualizations and a Multilevel Theoretical Model. Res. Occup. Stress Well Being.

[22] Peihang S., Zhang X., Sun Y., Ma H., Jiao M., Xing K., Kang Z., Ning N., Fu Y., Wu Q. (2017). Workplace Violence against Health Care Workers in North Chinese Hospitals: A Cross-Sectional Survey. Int. J. Environ. Res. Public Health.

[23] Schablon A., Wendeler D., Kozak A., Nienhaus A., Steinke S. (2018). Prevalence and Consequences of Aggression and Violence towards Nursing and Care Staff in Germany—A Survey. Int. J. Environ. Res. Public Health.

[24] Gutiérrez-Cobo M.J., Megías A., Gómez-Leal R., Cabello R., Fernández-Berrocal P. (2018). The Role of Emotional Intelligence and Negative Affect as Protective and Risk Factors of Aggressive Behavior: A Moderated Mediation Model. Aggress. Behav..

[25] Sun H., Wang S., Wang W., Han G., Liu Z., Wu Q., Pang X. (2020). Correlation between Emotional Intelligence and Negative Emotions of Front-line Nurses during the COVID-19 Epidemic: A Cross-sectional Study. J. Clin. Nurs..

[26] Hatfield E., Cacioppo J., Rapson R., Margaret S.C. (1992). Primitive emotional contagion. Current Directions in Psychological Science.

[27] Law K.S., Wong C.-S., Song L.J. (2004). The Construct and Criterion Validity of Emotional Intelligence and Its Potential Utility for Management Studies. J. Appl. Psychol..

[28] Yue Y., Wang K., Groth M. (2017). Feeling Bad and Doing Good: The Effect of Customer Mistreatment on Service Employee’s Daily Display of Helping Behaviors. Pers. Psychol..

[29] Cai D., Li F., Feng T., Liu B., Qi L., Men C. (2020). Mistreatment from Patients and Nurses’ Career Withdrawal Intention: Does Political Skill Matter?. Asia Pac. J. Hum. Resour..

[30] Şat S.Ö., Akbaş P., Yaman Ş. (2021). Nurses’ Exposure to Violence and Their Professional Commitment during the COVID-19 Pandemic. J. Clin. Nurs..

[31] Wickrama K., Lee S., Klopack E., Wickrama T. (2019). Stressful Work Conditions, Positive Affect, and Physical Health of Middle-Aged Couples: A Dyadic Analysis. Stress Health.

[32] Bendersky C., Brockner J. (2020). Mistreatment from Peers Can Reduce the Effects of Respectful Treatment from Bosses, and Respectful Peers Can Offset Mistreatment from Bosses. J. Organ. Behav..

[33] Miner K., Cortina L. (2016). Observed Workplace Incivility toward Women, Perceptions of Interpersonal Injustice, and Observer Occupational Well-Being: Differential Effects for Gender of the Observer. Front. Psychol..

[34] Hershcovis S., Barling J. (2009). Towards a Multi-Foci Approach to Workplace Aggression: A Meta-Analytic Review of Outcomes from Different Perpetrators. J. Organ. Behav..

[35] Labrague L., McEnroe-Petitte D. (2018). Job Stress in New Nurses during the Transition Period: An Integrative Review. Int. Nurs. Rev..

[36] Tee E. (2015). The Emotional Link: Leadership and the Role of Implict and Explicit Emotional Contagion Processes across Multiple Organizational Levels. Leadersh. Q..

[37] Ni X., Zhou H., Chen W. (2020). Addition of an Emotionally Stable Node in the SOSa-SPSa Model for Group Emotional Contagion of Panic in Public Health Emergency: Implications for Epidemic Emergency Responses. Int. J. Environ. Res. Public Health.

[38] May D.D., Grubbs L.M. (2002). The Extent, Nature, and Precipitating Factors of Nurse Assault among Three Groups of Registered Nurses in a Regional Medical Center. J. Emerg. Nurs..

[39] Du J., Fan X., Feng T. (2011). Multiple Emotional Contagions in Service Encounters. J. Acad. Mark. Sci..

[40] Pugh S. (2001). Service with a Smile: Emotional Contagion in the Service Encounter. Acad. Manag. J..

[41] Geßler S., Nezlek J., Schuetz A. (2020). Training Emotional Intelligence: Does Training in Basic Emotional Abilities Help People to Improve Higher Emotional Abilities?. J. Posit. Psychol..

[42] Gross J. (2002). Emotion Regulation: Affective, Cognitive, and Social Consequences. Psychophysiology.

[43] Waugh C.E. (2020). The Roles of Positive Emotion in the Regulation of Emotional Responses to Negative Events. Emotion.

[44] Hagger M., Koch S., Chatzisarantis N., Orbell S. (2017). The Common Sense Model of Self-Regulation: Meta-Analysis and Test of a Process Model. Psychol. Bull..

[45] Gabriel A., Diefendorff J. (2015). Emotional Labor Dynamics: A Momentary Approach. Acad. Manag. J..

[46] Li Y., Chen M., Lyu Y., Qiu C. (2016). Sexual Harassment and Proactive Customer Service Performance: The Roles of Job Engagement and Sensitivity to Interpersonal Mistreatment. Int. J. Hosp. Manag..

[47] Sultana R., Yousaf A., Khan I., Saeed A. (2016). Probing the Interactive Effects of Career Commitment and Emotional Intelligence on Perceived Objective/Subjective Career Success. Pers. Rev..

[48] Gross J. (1998). The Emerging Field of Emotion Regulation: An Integrative Review. Rev. Gen. Psychol..

[49] Podsakoff P., MacKenzie S., Lee J.-Y., Podsakoff N. (2003). Common Method Biases in Behavioral Research: A Critical Review of the Literature and Recommended Remedies. J. Appl. Psychol..

[50] Brislin R.W., Lonner W.J., Berry J.W. (1986). The wording and translation of research instruments. Field Methods in Cross-Cultural Research.

[51] Skarlicki D., Van Jaarsveld D., Walker D. (2008). Getting Even for Customer Mistreatment: The Role of Moral Identity in the Relationship between Customer Interpersonal Injustice and Employee Sabotage. J. Appl. Psychol..

[52] McBane D.A. (1995). Empathy and the Salesperson: A Multidimensional Perspective. Psychol. Mark..

[53] Alshowkan A. (2015). Nurses Attitude toward People with Mental Illness. Eur. Psychiatry.

[54] Lisa E., Anne S., Cassidy M. (2019). Age Differences in Negative, but Not Positive, Rumination. J. Gerontol. Ser. B..

[55] Aiken L., West S. (1991). Multiple Regression: Testing and Interpreting Interactions.

[56] Tang N., Thomson L. (2019). Workplace Violence in Chinese Hospitals: The Effects of Healthcare Disturbance on the Psychological Well-Being of Chinese Healthcare Workers. Int. J. Environ. Res. Public Health.

[57] Cheung T., Lee P., Yip P. (2017). Workplace Violence toward Physicians and Nurses: Prevalence and Correlates in Macau. Int. J. Environ. Res. Public Health.

[58] Yıldırım D. (2009). Bullying among Nurses and Its Effects. Int. Nurs. Rev..

[59] Wang M., Liao H., Zhan Y., Shi J. (2011). Daily Customer Mistreatment and Employee Sabotage Against Customers:Examining Emotion and Resource Perspectives. Acad. Manag. J..

[60] Isbell L.M., Boudreaux E.D., Chimowitz H., Liu G., Cyr E., Kimball E. (2020). What Do Emergency Department Physicians and Nurses Feel? A Qualitative Study of Emotions, Triggers, Regulation Strategies, and Effects on Patient Care. BMJ Qual. Saf..

[61] Totterdell P. (2000). Catching Moods and Hitting Runs: Mood Linkage and Subjective Performance in Professional Sport Teams. J. Appl. Psychol..

[62] Kim J. (2019). Emotional Labor Strategies, Stress, and Burnout Among Hospital Nurses: A Path Analysis. J. Nurs. Sch..

[63] Guenter H., van Emmerik I.J.H., Schreurs B. (2014). The Negative Effects of Delays in Information Exchange: Looking at Workplace Relationships from an Affective Events Perspective. Hum. Resour. Manag. Rev..

[64] Judge T., Scott B., Ilies R. (2006). Hostility, Job Attitudes, and Workplace Deviance: Test of a Multilevel Model. J. Appl. Psychol..

[65] Matta F., Erol T., Johnson R., Bıçaksız P. (2014). Significant Work Events and Counterproductive Work Behavior: The Role of Fairness, Emotions, and Emotion Regulation. J. Organ. Behav..

[66] Botsford Morgan W., Perry S.J., Wang Y. (2018). The Angry Implications of Work-to-Family Conflict: Examining Effects of Leadership on an Emotion-Based Model of Deviance. J. Vocat. Behav..

[67] Leiba P. (1992). Learning from Incidents of Violence in Health Care. An Investigation of ‘Case Reports’ as a Basis for Staff Development and Organisational Change. Nurse Educ. Today.

[68] Lin Y.-H., Liu H.-E. (2005). The Impact of Workplace Violence on Nurses in South Taiwan. Int. J. Nurs. Stud..

[69] Lee J., Jensen J. (2014). The Effects of Active Constructive and Passive Corrective Leadership on Workplace Incivility and the Mediating Role of Fairness Perceptions. Group Organ. Manag..

[70] Hsu C.-P., Chiang C.-Y., Chang C.-W., Huang H.-C., Chen C.-C. (2015). Enhancing the Commitment of Nurses to the Organisation by Means of Trust and Monetary Reward. J. Nurs. Manag..

[71] Huyghebaert T., Gillet N., Audusseau O., Fouquereau E. (2019). Perceived Career Opportunities, Commitment to the Supervisor, Social Isolation: Their Effects on Nurses’ Well-Being and Turnover. J. Nurs. Manag..

[72] Karatuna I., Jönsson S., Muhonen T. (2020). Workplace Bullying in the Nursing Profession: A Cross-Cultural Scoping Review. Int. J. Nurs. Stud..

